# Analysis of the translatome in solid tumors using polyribosome profiling/RNA-Seq

**DOI:** 10.14440/jbm.2016.151

**Published:** 2016-11-21

**Authors:** Pauline Adjibade, Valérie Grenier St-Sauveur, Arnaud Droit, Edouard W. Khandjian, Paul Toren, Rachid Mazroui

**Affiliations:** ^1^Centre de Recherche en Cancérologie. Centre de Recherche du CHU de Québec. Département de Biologie Moléculaire, Biochimie Médicale et Pathologie, Faculté de Médecine, Université Laval, Québec, PQ, Canada; ^2^Centre de Recherche du CHU de Québec. Département de Médecine Moléculaire, Faculté de Médecine, Université Laval, Québec, PQ, Canada; ^3^Centre de Recherche, Institut Universitaire en Santé Mentale de Québec. Département de Psychiatrie et de Neurosciences, Faculté de Médecine, Université Laval, Québec, PQ, Canada; ^4^Centre de Recherche du CHU de Québec. Département de Chirurgie, Faculté de Médecine, Université Laval, Québec, PQ, Canada.

**Keywords:** polyribosome profiling, RNA-Seq, xenografts, translatome

## Abstract

Gene expression involves multiple steps from the transcription of a mRNA in the nucleus to the production of the encoded protein in the cytoplasm. This final step occurs through a highly regulated process of mRNA translation on ribosomes that is required to maintain cell homeostasis. Alterations in the control of mRNA translation may lead to cell’s transformation, a hallmark of cancer development. Indeed, recent advances indicated that increased translation of mRNAs encoding tumor-promoting proteins may be a key mechanism of tumor resistance in several cancers. Moreover, it was found that proteins whose encoding mRNAs are translated at higher efficiencies may be effective biomarkers. Evaluation of global changes in translation efficiency in human tumors has thus the potential of better understanding what can be used as biomarkers and therapeutic targets. Investigating changes in translation efficiency in human cancer cells has been made possible through the development and use of the polyribosome profiling combined with DNA microarray or deep RNA sequencing (RNA-Seq). While helpful, the use of cancer cell lines has many limitations and it is essential to define translational changes in human tumor samples in order to properly prioritize genes implicated in cancer phenotype. We present an optimized polyribosome RNA-Seq protocol suitable for quantitative analysis of mRNA translation that occurs in human tumor samples and murine xenografts. Applying this innovative approach to human tumors, which requires a complementary bioinformatics analysis, unlocks the potential to identify key mRNA which are preferentially translated in tumor tissue compared to benign tissue as well as translational changes which occur following treatment. These technical advances will be of interest to those researching all solid tumors, opening possibilities for understanding what may be therapeutic Achilles heels’ or relevant biomarkers.

## BACKGROUND

mRNA translation is a highly controlled process. Protein synthesis relies on the regulation of mRNA synthesis and degradation as well as on its translation efficiency. Upon synthesis, mRNA undergoes multiple processing steps before being exported to the cytoplasm to be either stored in untranslated form, directed to degradation or loaded onto ribosomes for conversion into proteins [1-3]. Thus, mRNAs produced during transcription are not necessarily expressed during translation. Because of this limitation, the transcriptomic measurement of steady-state mRNA level by methods such as microarray or deep RNA sequencing (RNA-Seq) is helpful, but not sufficient to adequately profile the expression of mRNAs that is critical for cell homeostasis and whose alteration promotes development of human diseases including cancer and its resistance to either drugs or radiation treatments. Direct proteomic analyses often do not match with RNA-Seq data. However, proteomics approaches are also limited, particularly in their ability to assess changes on a global cellular scale. Identification of mRNA translation levels is likely to be relevant in cancer research, and can now be achieved by measuring which genes are preferentially translated during cancer development and resistance.

Indeed, upregulation of the translation of mRNAs encoding tumor-promoting proteins is a well-recognized mechanism of tumor progression [4,5]. Two important translational pathways, the mTORC1 and phosphorylation of eIF2α pathways, generally regulate translation within tumorigenesis. While mTORC1 activates translation of mRNAs encoding growth factors [6], the phosphorylation of the translation initiation eIF2α factor modulates translation under a variety of stimuli involved in cancer such as ionizing radiation and anticancer drugs, towards expression of resistance-promoting factors [7-9]. Additional mechanisms that allow efficient translation of mRNAs involve two well characterized cis-acting RNA elements present in the corresponding mRNAs encoding cancer-promoting functions: the IRESs (Internal Ribosome Entry Sites) [10-11] and uORFs (upstream Open Reading Frames) [7-9]. Modulation of translation that occurs during drugs treatment is also inferred to be critical for resistance, in part by inducing angiogenesis [12]. Many chemotherapeutic molecules have been selected owing to their potent anti-angiogenic effects. Treatment of tumors with anti-angiogenic chemotherapeutics induces loss of tumors vasculature which limits both nutrient and oxygen supplies, thus preventing tumor growth and survival [13]. Resistant tumor cells including cancer-stem cells can however survive these inadequate nutrient and oxygen accessibility stressful environments in part *via* activation of stress-based adaptive translational mechanisms [14,15]. Among these, phosphorylation of eIF2α through activation of upstream stress kinases (*e.g.*, HRI, GCN2 and PERK) is a major stress-induced event known to modulate mRNA translation during both hypoxia and nutrient deprivation towards the preferential expression of pro-survival functions [7-9, 16-18]. For example, the preferential translation of hypoxia-inducible factors (*e.g.*, HIF1) and downstream growth factors are responsible for angiogenesis, invasion and metastasis, inducing chemoresistance [15]. Thus, translation regulation, as a result of drugs treatment-induced tumors microenvironment alteration, contributes to the resistance of cancer cells to drugs treatment.

Global changes that occur in translation with the development of cancer are still not well defined and are not detectable with commonly performed DNA or RNA-Seq [19]. Polyribosome profiling coupled with RNA-Seq is a cutting-edge approach used to measure the translatome [4,5]. It relies on the purification of polyribosomes on sucrose gradients using ultracentrifugation followed by the separation of mRNAs based on the number of bound ribosomes [20-24], which reflects their translation efficiency [25,26]. Actively translated mRNAs are generally those associated with heavy polyribosomes (3-4 ribosomes or more) corresponding to fractions sedimenting at the bottom of the sucrose gradient. Polyribosome-associated mRNAs are isolated and identified by DNA microarray [27-29] and more recently with the more robust RNA-Seq technology [30]. Polyribosome profiling coupled with RNA-Seq [31-33] thus monitors the translational status of most processed mRNA in addition to measuring differences in the translation of alternative transcript isoforms [31,34]. In cancer, polyribosome profiling permits a detailed and global investigation into cellular and cancer biology. Specifically, it allows the identification of changes in translational efficiency which permit cancer cell survival following either treatment with radiation [35,36] or upon exposure to hypoxia [37,38] as well as translational changes which promote cell invasion and metastases [36]. For example, using polyribosomal profiling analyses of AR-negative PC3 cells, Hsieh *et al*. [39] found that the mTORC1-eIF4F translational axis drives a metastatic phenotype through preferential translation of mRNAs encoding proteins involved in cell invasion and metastases including YB1, MTA1 and CD44 [39,40], in keeping with preclinical data reporting abnormal activation of mTORC1 and mRNA translation [41]. Although limited in number, these recent seminal studies supported the powerful use of polyribosome profiling to generate genome-wide cancer translatome data in cell culture. Despite these advances, the use of this technique *in vivo* is currently in its infancy, mainly because of limitations arising from the poor quality of isolated tumors polyribosomes.

Polyribosomes are generally prepared by velocity sedimentation through linear sucrose density gradients of total cytoplasmic extracts. However, our initial experiments showed that this classical method is inefficient to prepare polyribosomes from tissues and tumors. Our optimized homemade protocol presented here uses an initial step of ultracentrifugation through a sucrose cushion that enriches polyribosomes [42], which are then fractionated through sucrose gradients. Through this protocol, we have successfully generated high-quality polyribosome profiles prepared from prostate cancer specimens derived from transurethral resection of the prostate as well as from human tumors grown in mice. Our polyribosomes preparation contains intact RNA that can be readily amplified and analyzed by standard techniques including RNA-Seq. The protocol we are presenting is thus suitable for quantitative analysis of translation that occurs in human tumor xenografts and patients samples.

## MATERIALS

### Reagents

Establishment of xenografts and collection of transurethral prostate samples:

• 25G needle syringe
• Sterile PBS (phosphate-buffered saline) (Wisent, cat. # 311-010-CL)
• Matrigel Membrane Matrix (Corning, cat. # 356234)
• 3M Vetbond Tissue adhesive
• Hank’s Balanced Salt Solution (Wisent, cat. # 311-512-CL)

### Sucrose solution preparation

• D-sucrose (molecular biology & density gradient) (Fisher scientific, cat. # BP220)
• Glycerol (Sigma-Aldrich, cat. # 49767)
• Tris Hydrochloride (Fisher scientific, cat. # BP153)
• NaCl (Bio Basic, cat. # DB0483)
• MgCl_2_ (Magnesium chloride hexahydrate) (Sigma-Aldrich, cat. # M2670)
• DL-Dithiothreitol (DTT) (Sigma-Aldrich, cat. # D9779)
• Nonidet P40 substitute (USB, cat. # 19628 500 ML)
• Complete protease inhibitor cocktail tablets (Roche, cat. # 11836170001)
• RNAseOUT Recombinant Ribonuclease Inhibitor (Life Technologies, cat. # 10777019)
• Sodium deoxycholate (Sigma-Aldrich, cat. # D6750)
• Bromophenol blue (Fisher scientific, cat. # B3925)
• Cycloheximide (10 mg/ml; Sigma-Aldrich, cat. DEPC (Diethylpyrocarbonate) (Sigma-Aldrich, cat. # D5758)

### RNA extraction

• Proteinase K (Bio Basic, cat. # PB0451)
• Acidic phenol (Phenol:Chloroform 5:1 pH 4.3–4.7, Sigma-Aldrich, cat. # P1944)
• Phenol:Chloroform:Isoamyl alcohol (PCI) (125:24:1) (Sigma-Aldrich, cat. Pure Ethanol and 75% Ethanol
• Glycogen (20 µg/µl; Life Technologies, cat. # 10814010)
• Isopropanol (Fisher scientific, cat. # A416P)
• EDTA (Fisher scientific, cat. # BP120)
• SDS (J.T. Baker, cat. # 4095)
• NaOAc (Sodium Acetate Trihydrate) (Bio Basic, cat. #SB0481)

### RNA-Seq

• Illumina TruSeq stranded mRNA sample preparation kit (Illumina Inc., San Diego, CA, USA)
• Agencourt AMPure XP beads (Beckman Coutler, Missisauga, Ontario, Canada)

### Recipes

Prepare solutions in RNAse-free glassware and plastic ware (if possible use individual wrapped plastic ware).

**NOTE**: Buffers and sucrose solutions (15%, 50% and 55%) should be preferentially prepared the day of the experiment.

• Buffer #1: Prepare a solution containing 20 mM Tris (pH 7.4), 150 mM NaCl, 5 mM MgCl_2_ in sterile distilled water. Store at 4ºC until use. Before use, add 1 mM DTT, complete protease inhibitor cocktail (1 final concentration) and 60 U/ml RNAseOUT.
• Buffer #2: Prepare a solution containing 20 mM Tris (pH 7.4), 150 mM NaCl, 5 mM MgCl_2_, 1% Nonidet P-40 substitute in sterile distilled water. Store at 4ºC. Before use, add 1 mM DTT, 1% sodium deoxycholate, 50 µg/ml cycloheximide, complete protease inhibitor cocktail (1 final concentration) and 60 U/ml RNAseOUT.
• 15%, 50% and 55% sucrose solutions: Dissolve 15 g, 50 g or 55 g of sucrose (for 15%, 50% and 55% sucrose solutions respectively) in 40 ml of sterile distilled water supplemented with 20 mM Tris (pH 7.4), 100 mM NaCl and 3 mM MgCl_2_. Mix until complete dissolution and complete to 100 ml with sterile distilled water. Store at 4ºC until use. Before use, add 1 mM DTT, 50 µg/ml cycloheximide, complete protease inhibitor cocktail (1 final concentration) and 8 U/ml RNAseOUT.
• 60% sucrose solution: Dissolve 60 g of sucrose in 40 ml of sterile distilled water supplemented with 10% glycerol (v/v), 20 mM Tris (pH 7.4), 100 mM NaCl and 3 mM MgCl_2_. Mix until complete dissolution and complete to 100 ml with sterile distilled water. Add a little bit of bromophenol blue in order to get a dark coloration and agitate until the solution is homogeneous. Store at 4ºC until use.
• RNA extraction buffer: Prepare a solution containing 10 mM Tris (pH 7.4), 1 mM EDTA (pH 8.0), 0.2% SDS and 0.8 mg/ml proteinase K in sterile distilled water.
• NaOA_c_ 3M, pH 5.2: Dissolve 204.12 g of sodium acetate trihydrate in 400 ml of sterile distilled water. Adjust to pH 5.2 with acetic acid. Complete to 500 ml with sterile distilled water. Sterilize by autoclaving and store at room temperature.
• Preparation of DEPC-treated water: Mix 1 ml of DEPC with 1 L of water, stir overnight and then autoclave.

**CAUTION**: DEPC causes irritation to eyes, skin and mucous membranes. It is suspected to be a carcinogen. Prepare the solution in a fume hood and wear gloves. Alternatively, DEPC-treated water is also available on the market, *e.g.*, Invitrogen, cat. # 750023.

### Equipment

• Dounce tissue homogenizer with teflon pestle, working volume 7 ml
• Beckman SW 40 Ti rotor and swinging buckets (Beckman Coulter, Fullerton, CA)
• Tubes PA Thinwall 12 ml (Fisher scientific, cat. # 03699)
• ISCO Density Gradient Fractionation System with UA-6 detector (Brandel, Gaithersburg, MD/Isco, Inc., Lincoln, NE)
• Heating blocks
• Refrigerated benchtop centrifuge
• Spectrophotometer (Eppendorf BioPhotometer)
• HiSeq 2500 system (Illumina Inc., San Diego, CA, USA)

## PROCEDURE

1. Establishment of xenografts: Our protocol for the establishment of the human prostate tumor xenografts uses procedures and conditions approved by the Laval University’s Animal Care Committee.
  1.1. Harvest LNCaP prostate cancer cells by trypsinization then inactivate trypsin by adding media containing 10% FBS and centrifuge for 5 min at 1500 rpm at room temperature.
  1.2. Remove the supernatant and wash the cell pellet twice with PBS.
  1.3. Resuspend the cell pellet in PBS.
  1.4. Count the cells with a Hemocytometer to determine cell concentration.
  1.5. Centrifuge the cell suspension for 5 min at 1500 rpm at room temperature.
  1.6. Discard the supernatant and resuspend the cell pellet at 2 10^6^ cells/100 in a Matrigel matrix: PBS (1:1) mix.
**NOTE**: From this step, always keep working on ice otherwise the Matrigel matrix could start polymerize. The Matrigel matrix is previously thawed overnight at 4ºC.
  1.7. Prepare one tube per mouse containing 250 of the cell suspension and keep them on ice. Anesthetize each mouse with isoflurane inhalant just prior injection. Carefully mix the cell suspension by up and down to prevent cells from settling and inject, with a 25G needle, 100 of the cell suspension (2 × 10^6^-day old nude female mice. Add a drop of tissue adhesive over the injection site before pulling out the needle in order to avoid losing material. The tumor growth is monitored at least twice a week by using calipers for measuring the size of the tumors.
**TIP**: Agitate the cell suspension prior to inoculation to prevent the cells from settling. The injection site and animal well-being is monitored according to institutional standards.
2. Preparation of extracts from human xenografts and tumors: Euthanasia of mice was conducted according to procedures approved by the Laval University’s Animal Care Committee.
  2.1. Collection of human xenografts:
    2.1.1. When tumors reached approximately 150–200 mm^3^ in size, mice are sacrificed. Tumors are collected and washed with PBS to eliminate murine blood.
    2.1.2. Dry the tumors with a paper towel and transfer them into a 1.5 ml or a 15 ml tube according to the size of the tumors.
    2.1.3. Snap-freeze in liquid nitrogen and store the tumors at −80°C until the day of the experiment.
  2.2. Collection of transurethral prostate samples: Following transurethral resection using monopolar cautery, prostate chips are collected on ice and brought to the pathology department. According to the pathologist, portions are then released for research with the rest undergoing routine pathologic evaluation. Prostate chips are rinsed with ice cold HBSS, weighed and frozen in liquid nitrogen, with 0.5–1 g per cryotube. The delay from resection to storage ranges from 45 to 90 min.
  2.3. Homogenization of the extracts: From this step, all the manipulations are carried out on ice. The day of the experiment, thaw the samples on ice for 5–10 min. Transfer one tumor sample in an ice-cold Dounce Tissue homogenizer (RNAse-free) containing 2 ml of buffer #1.
**NOTE**: Xenografts weight is between 0.1 and 0.8 g. Human biopsies weight is between 0.5 and 0.8 g.
  2.4. Homogenize the tumors on ice with 10 strokes of the pestle of the ice-cold Dounce tissue homogenizer. Put a drop of the homogenate onto a microscope slide and check for cell lysis by phase-contrast microscopy. Only nuclei are visible after a complete cell lysate.
**CAUTION**: Keeping samples and solutions on ice throughout all manipulations is important to reduce RNAse activity. Avoid harsh homogenization which may induce destabilization of polyribosomes.
  2.5. Clarification of the homogenate: Centrifuge the homogenate at 12000 rpm (13500 g) for 15 min at 4°C. Transfer the supernatant into a new 15 ml Falcon tube. Adjust the volume of the supernatant to 8 ml with the buffer #2.
**TIP:** Measure of RNA concentration with a spectrophotometer may be done to estimate the quantity of RNA obtained. One OD_260_ unit correspond to 40 µg/ml of RNA.
**NOTE**: Keeping the tumors cold helps in preventing fortuitous dissociation of polyribosomes and to prevent mRNA degradation during the preparation of the extracts. For optimal polyribosome profiles results, use cycloheximide, an antibiotic that block translation elongation by stalling polyribosomes-RNA interactions.
3. Preparation of the density gradient fractionation system and sucrose gradients
  3.1. Priming the tubing system: Wash the tubing system with 0.1% SDS for 5 min, with DEPC-treated water for 5 min and pump air in order to dry the apparatus.
  3.2. Preparing the gradients: Set the gradient parameters in order to obtain a linear 15%–55% gradient of 11 ml according to the manufacturer’s instructions.
**TIP**: During the preparation of the gradients, ensure no air bubbles are trapped inside the tubing system, since bubbles disturb the gradient formation. When the gradient formation is finished, carefully transfer the tube on ice without disturbing it.
4. Loading the extracts onto sucrose gradients and ultracentrifugation
  4.1. Prepare a 3 ml cushion of 50% sucrose in a 12 ml polycarbonate ultracentrifuge tube by pipetting the sucrose solution against the tube wall.
  4.2. Load the supernatant (step 2.5) onto the 50% sucrose cushion and centrifuge for 2 h at 35000 rpm (200000 *g*) at 4ºC.
**NOTE**: Through this critical step, polyribosomes are enriched and non polyribosomal complexes that do not penetrate the sucrose cushion are removed [42].
**TIP**: As with this step and subsequently ones, placing the ultracentrifugation rotor and the buckets in the cold room (4ºC) is recommended until ready to use. To load the supernatant, place the tip of the pipette against the tube wall close to the surface of the sucrose cushion without disturbing it. Ensure all loaded tubes are equivalent weights with a maximal difference of 0.01 g. Buffer #2 can be added to the top of the gradients to balance tubes weights.
  4.3. Remove all the sucrose after centrifugation and resuspend the resulting translucent pellet in 1 ml of buffer #2 by pipetting up and down.
**NOTE:** We do not recommend to vortex your extract.
  4.4. Incubate on ice for 30 min to complete homogenization.
**TIP**: Mix the extract by repeated pipetting during the incubation in order to obtain a complete resuspension of the pellet.
  4.5. Estimate the RNA concentration of the resuspended extract by measuring the OD_260_ with a spectrophotometer.
**NOTE**: Proteins as well as non-ionic detergents will also absorb UV light near 260 nm and this will result in an overestimation of RNA concentration. All UV blank determination should be done using buffer #2 as standard. At this step, only an estimation of RNA concentration is needed.
  4.6. Carefully load the sample (between 10–20 OD_260_) on the top of the 15%–55% sucrose gradient (prepared at step 3.2) without disturbing the interface.
**CAUTION**: This step is critical in order to maintain polyribosomes integrity and to obtain good profiles. An option is to pipet gently against the wall of the tube, near the surface of the gradient.
**TIP**: Ensure all loaded tubes are equivalent weights with a maximal difference of 0.01 g. Buffer #2 can be added to the top of the gradients to balance them.
  4.7. Gently place the gradients in a Beckman SW 40 Ti rotor and centrifuge the gradients at 37000 rpm (230000 *g*) for 2 h 30 min at 4ºC.
  4.8. Following centrifugation, remove the tubes carefully in order to not disturb the gradients and place them at 4ºC.
**NOTE**: Alternatively, you can place the gradient in a pre-formed hole in the ice made previously with an empty tube.
5. Fractionation of polyribosomes
  5.1. Turn on the ISCO UA-6 detector to allow the UA-6 to warm up 30–45 min.
  5.2. Set the baseline by using a 15% sucrose solution. Refer to the manufacturer’s manual for more information.
  5.3. Set the following parameters on the apparatus: (1) Sensitivity: 0.2 (tumor biopsies) or 0.5 (xenograft); (2) Peak separator: OFF; (3) Noise Filter: 1.5.
**TIP**: The sensitivity of the detector can be changed depending of the quantity of sample. The less material you have, the higher sensitivity of the detector should be chosen.
  5.4. Reading the gradients:
    5.4.1. Pump the 60% sucrose solution (colored with bromophenol blue) into the tubing system until it reaches the needle.
**TIP**: Make sure to have a few drops dripping out of the needle. This will minimize the risks of introducing air bubbles into the gradient.
    5.4.2. Place carefully the gradient into the tube piercer of the Automated Density Fractionation System and then pierce the tube with the needle.
**TIP**: Be sure to not create any waves in the gradient when you install the gradient in the tube piercing system.
    5.4.3. Start introducing the 60% sucrose solution into the gradient, set the chart speed to 60 and start the recording program.
  5.5. Collect each fraction (approximately 500 ) in an individual pre-labelled Eppendorf tube while the polyribosome profile is recorded on the chart paper.
**NOTE**: The Optical Unit of the Density Gradient Fractionation System is configured to read the absorbance at 254 nm. Alternatively, an electronic acquisition of the polyribosome profile can be obtained.
  5.6. At the end of each run, transfer the collected fractions on ice. At this time, either store collected fractions at−80°C or directly precipitate protein-RNA complexes as follows.
**NOTE**: Typically, we collect 24 fractions of approximately 500 per gradient.
  5.7. Repeat steps 5.4 to 5.6 if there is more than one gradient to analyze.
**CAUTION**: Clean the tubing system between each gradient by pumping SDS 0.1% (5 min) and then DEPC-treated water (5 min). Ensure all water is out of the tube before the next gradient is processed.
6. RNA extraction and analysis
  6.1. Precipitate RNA-protein complexes by adding 3 volumes of pure cold ethanol to each collected fraction and incubate overnight at −20 °C.
  6.2. Centrifuge RNA-protein precipitates at 13000 rpm (16000 *g*) for 30 min at 4 °C, remove the supernatant and let the pellets air dry for 10 min.
  6.3. Pool the fractions corresponding respectively to light and heavy polyribosomes and resuspend the RNA-protein precipitate in 500 of RNA extraction buffer.
**TIP**: To prepare each pool, resuspend by pipetting up and down the pellet of the first fraction and then transfer the sample to the next fraction, *etc*.
  6.4. Incubate 20 min at 37ºC in a block heater.
  6.5. Add 500 of acidic phenol (5:1) pH 4.3–4.7.
  6.6. Vortex during 15 s and centrifuge at 13000 rpm (16000 × g) for 15 min at 4ºC. Transfer the aqueous phase to a new Eppendorf tube without disrupting the white middle interphase.
**TIP**: Avoid contamination with the organic phase.
  6.7. Add 500 of PCI (Phenol: chloroform: Isoamyl alcohol (125:24:1)) to the aqueous phase, vortex then centrifuge at 13000 rpm (16000 *g*) for 15 min at 4ºC.
  6.8. Transfer the aqueous phase in a new Eppendorf tube then add 2 volumes of pure EtOH, 1/10 volume of NaOAc 3M pH 5.2 and 1 of glycogen 20 µg/µl. Let precipitate overnight at −20ºC.
  6.9. Centrifuge at 13000 rpm (16000 × *g*) for 30 min at 4ºC. Carefully aspirate the supernatant without disturbing the pellet. Wash the pellet with 500 cold EtOH 70%. Centrifuge at 13000 rpm (16000 *g*) for 30 min at 4ºC. Carefully aspirate the supernatant and let the pellets air dry for few minutes.
**TIP**: Do not overdry the pellets because it will make it difficult to be totally resuspended.
  6.10. Resuspend the RNA pellet into a small volume (20 ) of RNAse free water.
**NOTE**: Alternatively, RNAstableBiomatrica, San Diego, CA, USA) may be used during long-term storage to protect samples from degradation.
7. RNA-Seq analysis: This protocol is based on that used by the Next-Generation Sequencing Platform, Genomics Center, CHU de Québec-Université Laval Research Center, Québec City, Canada
  7.1. Check RNA quality using a 2200 TapeStation system (Agilent Technologies, Santa Clara, CA, USA).
**CAUTION**: High quality RNA is essential for successful cDNA library. It is critical to check RNA integrity to ensure that differential RNA degradation of samples is not later mistaken for differential expression. The RNA integrity number (RIN) is used to assess RNA quality and a RIN higher of 7 is ideal for RNA-Seq analysis. RNA purity is determined by measuring the 260/280 and 260/230 ratios. Acceptable 260/280 ratio for RNA purity: > 1.8.
**TIP**: A minimum of 500 ng of total RNA is recommended for mRNA sequencing library preparation. However, some sequencing platforms can prepare cDNA library with less amount of total RNA (*e.g.*, 100 ng).
  7.2. Prepare mRNA sequencing libraries using the Illumina TruSeq stranded mRNA sample preparation kit in accordance with manufacturer’s instructions.
    7.2.1. Poly(A) RNA are isolated from total RNA using oligo-dT attached magnetic beads followed by fragmentation of mRNA. Alternatively, ribosomal RNA can be depleted from polyribosomal RNA using Ribo-Zero rRNA Removal kit (Illumina, San Diego, CA).
    7.2.2. The fragmented mRNA is used as template for cDNA synthesis by reverse transcription with random primers. The resulting cDNA are then converted into double-stranded cDNA that are end-repaired to incorporate the specific index adaptor for multiplexing.
    7.2.3. The cDNA library is further purified with Agencourt AMPure XP beads and amplified by PCR.
**NOTE:** The quality of final amplified libraries is examined with a DNA screentape D1000 on a TapeStation 2200. The quantification is done on the QBit 3.0 fluorometer (Thermo Fisher Scientific, Canada) as well as by qPCR using KAPA library quantification kits. (Kapa Biosystems, Wilmington, MA, USA).
  7.3. Sequencing and analysis: RNA-Seq libraries with unique index were pooled together in equimolar ratio. The pool is then sequenced using an HiSeq 2500 system at the Next-Generation Sequencing Platform, Genomics Center, CHU de Québec-Université Laval Research Center, Québec City, Canada, and data analyzed using standardized pipeline, from sample demultiplexing, quality control and trimming to transcript quantification and different expression analysis.

## ANTICIPATED RESULTS

**Figure 1.**
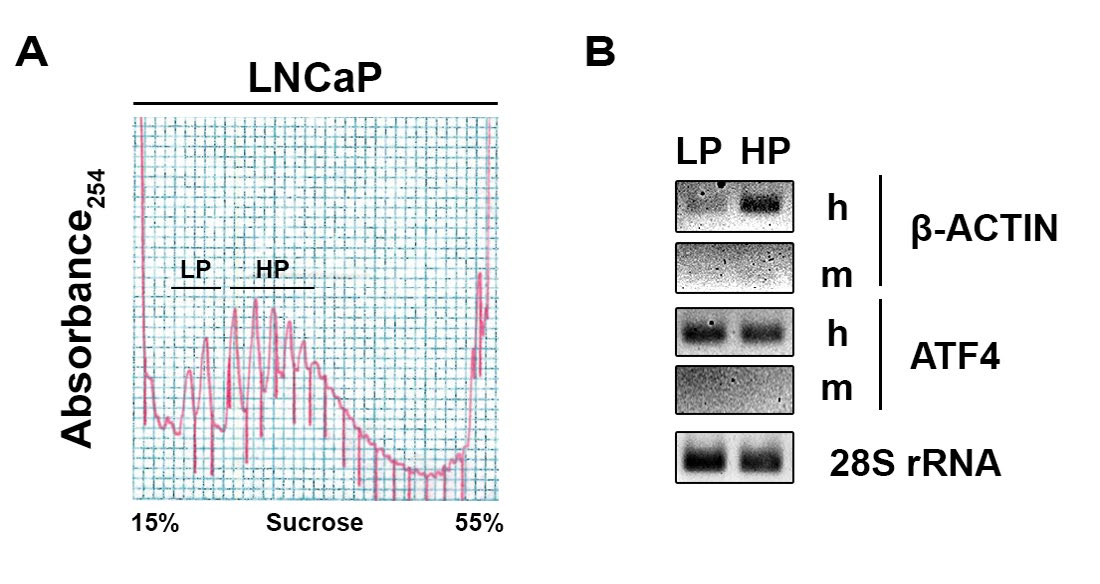
**Representative polyribosome profiles obtained from xenografts samples and RT-PCR of polyribosomal RNA. A.** Collected xenografts were homogenized and clarified by centrifugation. The resulting extracts were loaded onto a 50% sucrose cushion and centrifuged at 35 k rpm for 2h. The polyribosome-enriched resultant pellet was then resuspended and loaded on a 15%–55% sucrose gradient and the polyribosome profile was obtained by continuous UV (absorbance at 254 nm) monitoring during unloading. RNA was extracted from pooled fractions and verified for quality and integrity by a TapeStation 2200 (Agilent Technologies, Santa Clara, CA, USA), then analyzed by RT-PCR. LP: Light polyribosomes; HP: Heavy polyribosomes. **B.** Semi-quantitative RT-PCR of purified RNA from LP and HP using specific oligos for either human (h) β-ACTIN or ATF4, mouse (m) β-ACTIN or ATF4, and 28S rRNA. The control oligos, specific to mβ-ACTIN or mATF4, fail to amplify any products, attesting that our polyribosomes preparations are free of murine cross contamination. As expected, the results show that β-ACTIN mRNA is highly enriched in HP as compared to LP indicating efficient translation of this mRNA. On the contrary, the mRNA encoding ATF4 is moderately enriched in HP as compared to LP in keeping with the normal low expression of this regulatory gene, as compared to the high expression of housekeeping genes such as β-ACTIN.

**Figure 2.**
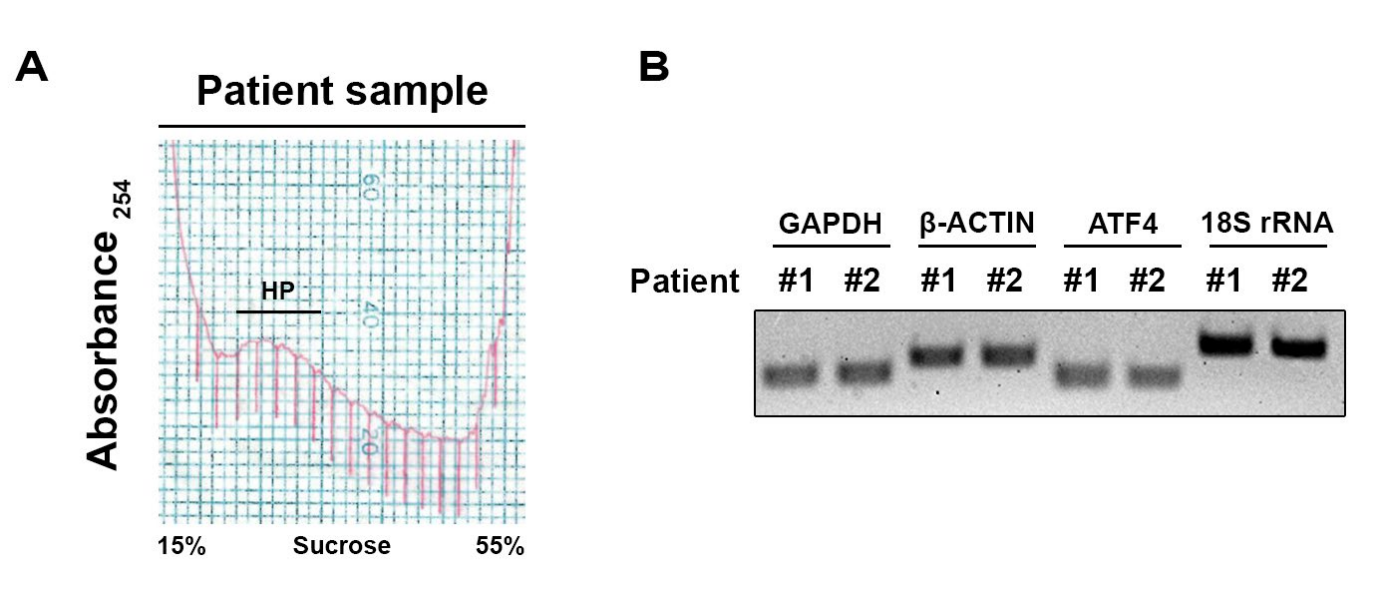
**Representative polyribosome profile obtained from prostate transurethral resection and RT-PCR of polyribosomal RNA. A.** Prostate tissue samples were collected and homogenized. The homogenates were then sedimented on a 50% sucrose cushion. The polyribosome-enriched resultant pellet was resuspended and loaded on a 15%–55% sucrose gradient and the polyribosome profile was obtained by continuous UV monitoring at 254 nm during fractionation of the gradient. RNA was extracted from pooled heavy polyribosomes (HP), and its quality and integrity was validated to be suitable for RNA-Seq as above. **B.** RNA isolated from HP of cancerous (#1) and benign (#2) prostate specimens was then analyzed by qRT-PCR using oligos specific to GAPDH, β-ACTIN, ATF4 and 18S rRNA. The amplified PCR products were then migrated on an agarose gel.

As shown in **Figure 1** and **2**, we have successfully generated high-quality polyribosome profiles prepared from human tumors grown in mice (**Fig. 1**) as well as from prostate cancer specimens derived from transurethral resection of the prostate (**Fig. 2**). This protocol also allows us to prepare intact polyribosome-bound mRNA which can be readily amplified and analyzed by RNA-Seq for further bioinformatics analyses as described [43]. By multiplexing 6 samples per HiSeq lane, our protocol should lead to approximately 100 M paired-end reads per sample. Through quantifying and comparing translational changes between benign tissue and in various stages of cancer progression and with resistance, this approach offers a unique opportunity to identify what are the key mRNAs preferentially translated in tumors as compared to benign tissues and following treatment resistance.

An important step of the identification of relevant translatome cancer data relies on an adapted bioinformatics analysis, which requires a well-developed pipeline dedicated to analyzing RNA-Seq data derived from human tumor polyribosome profiles. This includes poor quality sequence trimming, clipping of adapter sequences from the reads using softwares such as Trimmomatic. The resulting reads will be pseudo-aligned by bootstrap using Kallisto [44] to human transcriptome of the corresponding tissue (*e.g.*, prostate) and then analyzed for differential translational changes between tumors and benign specimens with Sleuth. Sleuth will use bootstrapping results from Kallisto to error-correct and evaluate significant differential expressions through model fitting (Wald test). The obtained translational variations will also be corrected for differences in the cytoplasmic level of mRNAs in order to identify translatome variations that are not due to alterations in the steady-state level of mRNAs [28,45]. Bioinformatic methods applied to score translation efficiency in a genome-wide manner are also described in recent reviews [45] and papers [43].

Polyribosome RNA-Seq does not generate however direct evidence of mature proteins [46]. In addition, some polyribosomal-associated mRNAs may not be translated owing to specialized translational regulatory mechanisms (*e.g.*, stalling [47]- and miRNA [4]-mediated inhibition of translation elongation) that blocks translation of target mRNAs despite their association with polyribosomes. Therefore, additional validation of translatome data should be obtained by combining polyribosomes profiling with proteomics approaches such as the innovative puromycin-associated nascent chain proteomics (PUNCH-P) complementary approach [48-50]. We select PUNCH over other proteomics approaches as it allows direct measurement of system-wide protein synthesis without the need of metabolic labeling in whole cells, and therefore it can be used to measure newly synthesized proteins in tumor tissues.

Validated corresponding genes whose mRNA translational efficiencies are significantly upregulated in tumors can be grouped in biological functional categories according to annotations from the gene ontology consortium [51]. Generated lists are then clustered using functional annotations from the gene ontology consortium [51] and compared to common oncogene and cancer specific lists. Subsequent identification of biological pathways and specific signatures differentially expressed can be achieved *via* an enrichment analysis on lists of up- and down-regulated genes (FDR < 0.05) using pathway analysis tools: the Kyoto Encyclopedia of Genes database. Prioritization for further validation and functional studies can be based on literatures, magnitude of translational changes and putative function, such as genes encoding functions relevant to tumors progression and resistance including cell cycle regulators, stress genes, DNA repair and cell survival. Standard *in vitro* (*e.g.*, CRISPR-CAS9 knockout) and *in vivo* (*e.g.*, mice xenografts) validation experiments of the identified targets should further confirm changes relevant to cancer and resistant phenotype, including proliferation, apoptosis or invasion assays, and tumor development, relevant to the putative function of the target gene. Subsequent patient validation studies will include evaluation of selected targets in the collected human tumors samples to confirm the tumor-specific nature of the candidate targets by immunohistochemistry or western blot. Further validation of the prognostic importance of these proteins may also be evaluated in accessible specific cancer microarrays.

In conclusion, applying polyribosomes profiling in human tumors may open possibilities for understanding what may be novel therapeutic Achilles heels’ for cancer cells or relevant biomarkers of treatment resistance. As aforementioned, the use of such an approach in prostate cancer cells has highlighted the role of YB-1 and MTA1 levels, with validation in human microarrays [40]. However, the possibility to directly analyze sensitive and resistant prostate tumors opens greater opportunities to identify key proteins useful as biomarkers or therapeutic targets against resistant prostate cancer. Finally, when applied to other cancer sites, this discovery strategy has clear potential to advance our fundamental knowledge and to identify therapeutic targets for treatment of other cancers.

The main limitation of our technique is that the amount of tissue required exceeds that typically available from percutaneous biopsies. Further, there is inevitable exposure to body and room temperature as part of the surgical procedure, as well as cautery artifact. Nonetheless, our results with transurethral-obtained prostate chips were reproducible and suggest the technique will be easily adaptable to other tumor sites. As compared to other proteomics approaches, polyribosome profiling is costly due to the expenses of RNA-Seq which however should drop with development of new generations of RNA-Seq. Although proteomics approaches appear less time consuming, necessitating less steps than polyribosome profiling to be completed, they are limited in their ability to cover the full proteome, and therefore in their ability to assess changes on a global cellular scale. Further, protein measurements with proteomics approaches reflect both protein synthesis and degradation. The latter is a highly regulated process that precludes direct assessment of translation efficiency which depends mainly on the rate of protein synthesis. Polyribosome profiling allows direct measurement of translation efficiency, circumventing possible contributions of protein degradation which could compromize the accuracy of the data. Finally, unlike protein, RNA molecules can readily be amplified, making their measurement for identification of novel isoforms as well as for comprehensive global analysis based on limited clinical samples more feasible. Similarly, evaluation of relative differences in protein levels following the development of resistance is more difficult with proteomics approaches [4,52]. However, suitable proteomic approaches such as PUNCH can be used to complement and to validate polyribosome profiling data.

**Table 1.** Troubleshooting table.

Step	Problem	Possible reason/cause	Solution/suggestions
4.5	Low concentration of RNA in the homogenate	• The pellet is not completely resuspended	• Resuspend again by pipetting up and down and incubate on ice for 10–15 min
5.5	**Low polyribosome peaks** are observed	• Degradation of RNA • “Runoff” of ribosomes	• Increase the quantity/concentration of RNAse inhibitors • Keep the temperature low (0–4); Keep all the solutions and materials cold and RNAse free • Tumors must be cooled/frozen as quickly as possible after harvesting and stored at –80; Keep all solutions cold and work on ice
5.5	Nothing is detected in the polyribosome profiles	• Polyribosome extraction didn’t work properly • Poor quality of the sample • Not enough tumor tissue material was used • Sensitivity is too low	• Increase the number of strokes during the mechanical lysis (add one or 2 more strokes) • Tumors must be cooled/frozen as quickly as possible after harvested and stored at –80 • For optimal polyribosomal profiles results, it is recommended to load a 10–20 OD_260_ • Increase the sensitivity of the UA-6 absorbance monitor by changing the setting to a higher sensitivity
5.5	Poor quality profiles	• Disrupted gradients	• The gradients should be handled with caution in order to minimize any perturbation

This protocol highlights a method to obtain polyribosomes profiles from solid tumors to facilitate global analysis of translational changes in solid tumors. Such analysis has a clear potential to advance our fundamental knowledge in cancer research. As it has previously been done with transcriptomic and proteomic data, the unique comprehensive data obtained may be translated to benefit patients through the identification of novel targets or biomarkers.

## TROUBLESHOOTING

Possible problems and their troubleshooting solutions are listed in **Table 1**.

## Abbreviation used

DTT: DL-dithiothreitol
HBSS: Hank’s balanced salt solution
PCI: Phenol:Chloroform:Isoamyl
PUNCH: puromycin-associated nascent chain
RIN: RNA integrity number
RNA-Seq: RNA sequencing
RT-PCR: reverse transcription polymerase chain reaction
